# Physiological effects of combined NaCl and NaHCO_3_ stress on the seedlings of two maple species

**DOI:** 10.3389/fpls.2023.1209999

**Published:** 2023-07-11

**Authors:** Bo Xu, Lina Cao, Zhenxing Zhang, Xinyu Li, Xiangyu Zhao, Xinyue Wang, Yining Wang, Bingchen Wu, Weihua Zhou, Chenlu Lin, Yufu Gao, Liping Rong

**Affiliations:** ^1^ College of Agriculture, Yanbian University, Yanji, China; ^2^ State Environmental Protection Key Laboratory of Wetland Ecology and Vegetation Restoration, Northeast Normal University, Changchun, China; ^3^ Key Laboratory of Vegetation Ecology, Ministry of Education, Jilin Songnen Grassland Ecosystem National Observation and Research Station, Northeast Normal University, Changchun, China

**Keywords:** cell membrane permeability, osmotic regulators, antioxidant enzyme, chlorophyll fluorescence, maple

## Abstract

Salt stress impacts growth and physiological processes in plants, and some plants exposed to salt stress will produce physiological mechanisms to adapt to the new environment. However, the effects of combined NaCl and NaHCO3 stress on the seedlings of Acer species are understudied. In this study, we designed an experiment to measure physiological characteristics by establishing a range of NaCl and NaHCO_3_ concentrations (0, 25, 50, 75, and 100 mmol L^-1^) to estimate the compound salt tolerance of *Acer ginnala* and *Acer palmatum*. When the concentrations of NaCl and NaHCO_3_ were 25 mmol L^-1^, the leaf water content, relative conductivity, malondialdehyde (MDA) content, proline content, soluble sugar content, and chlorophyll did not change (*p* > 0.05) in two maple seedlings. At concentrations greater than 50 mmol L^-1^, the relative conductivity and MDA content increased, proline and soluble sugars accumulated, and the potential activity of PS II (*F_v_/F_o_*), potential photochemical efficiency of PS II (*F_v_*/*F_m_*), PS II actual photochemical efficiency (Yield), and photosynthetic electron transfer efficiency (ETR) decreased (*p* < 0.05). The superoxide dismutase (SOD) and catalase (CAT) activities showed the same trend of first increasing and then decreasing (*p* < 0.05). The peroxidase (POD) activity increased only when concentrations of NaCl and NaHCO_3_ were 100 mmol L^-1^, while there was no statistical difference between the other treatments and the control. Therefore, the two maple seedlings adjusted their osmotic balance and alleviated oxidative stress by accumulating proline, soluble sugars and increasing CAT and SOD activities. Further analysis showed that both species are salt tolerant and the salt tolerance of *Acer ginnala* is better than that of *Acer palmatum*.

## Introduction

At present, soil salinization has become an increasingly severe ecological and environmental problem worldwide (Ustun et al., 2018; Litalien and Zeeb, 2020). The growth and development of plants in saline land was affected by destroying chloroplast structures and cell membranes (Zhuang et al., 2019), weakening of photosynthetic capacity (Yang et al., 2020), causing various metabolic disorders (Forni et al., 2017). Some plants exposed to salt stress will produce physiological mechanisms to adapt to the new environment (De Oliveira et al., 2022). However, the salt tolerance mechanism of plants is very complicated. Therefore, studying the physiological and ecological characteristics in the response to salt stress can help understand the sensitivity and resistance of plants and provide useful information for further introduction and cultivation in saline soil areas. In recent years, some scholars have studied the effects of salt stress on plants, but most focused on field crops (Thi Thu et al., 2020) and horticultural crop (Miao et al., 2020; Zhang et al., 2020). Research indicates that *Tamarix hispida* has the potential to remediate soil saline-alkali (Xie et al., 2023). *Catalpa bungei* mainly resists from saline-alkali stress by accumulating contents of soluble sugars and proline improving SOD enzymatic activity and photosynthesis (Gao et al., 2023), *Quercus chenii* could adapt to NaCl stress with a concentration below 3‰, and this concentration could be used as the critical reference concentration for the promotion of *Q. chenii* seedlings in the coastal regions of Jiangsu (Liu et al., 2023).

Many studies showed that trees played a very important role in the transformation of saline-alkali land (Xu et al., 2020). Therefore, it is necessary to focus studies of salt tolerance on trees and their related physiological characteristics to improve the utilization of saline-alkali land.

Many species of the *Acer* genus are important decorative trees throughout the world because of their attractive leaf colours and shapes (Wada and Ribbens, 1997). *Acer ginnala* and *Acer palmatum* are widely distributed species, mainly found in Japan, Korea, Mongolia, East Russia, and China (Dong et al., 2019). They not only have a high ornamental value but also economic value. There were some studies on the physiological characteristics of salt stress (Li et al., 2020), however, these studies mainly focused on the physiological response to different concentrations of NaCl. In the natural environment, the salts in soil are mainly neutral (NaCl and Na_2_SO_4_) and alkaline (Na_2_CO_3_ and NaHCO_3_) (Yin et al., 2019). Different types of salt stress have different effects on plant growth and physiology. Some scholars have studied the effects of mixed stress with Na_2_CO_3_ and NaHCO_3_ on the seed germination, seedling growth, and physiological characteristics of *Clematis* (Zhang et al., 2022) and *Chenopodium quinoa* (Wang et al., 2021). However, there are no reports yet on the response of maple trees to mixed saline-alkali stress with Na_2_CO_3_ and NaHCO_3_.

The purpose of this study was to clarify the physiological effects of combined NaCl and NaHCO_3_ stress on the seedlings of two maple species and evaluate the salt tolerance of two maples. The study results have important scientific significance and practical value in understanding the adaptability of maple trees, improving the utilization value of saline-alkali land, and guiding the planting and management of maple trees.

## Materials and methods

### Plant materials

*Acer ginnala* and *Acer palmatum* were used as the experiment material and were grown in Yanji city, Jilin Province, China (42°55’19” N, 129°29’20” E, and altitude 256 m above MSL). Seedlings (mean heights of 80 cm and ground diameters of 1 cm) were selected and transplanted into plastic pots (top diameter of 40 cm, bottom diameter of 35 cm) filled with a mixture of peat soil, sand, and solid manure (volume ratio 2:1:1). Each pot contained one single plant. After transplantation, the seedlings were watered adequately with ½-strength Hoagland solutions every morning between 09h00 and 10h00. There were five treatments and there was a total of 75 pots in the experiment, which included three replicates per treatment and five plants per replicate for each maple species. All potted plants were marked and placed randomly. We started the experiment two weeks after the saplings were transplanted.

### NaCl and NaHCO_3_ mixed treatments

A ½ Hoagland nutrient solution with different concentrations of mixed solution was prepared with equivalent molar concentrations of NaCl and NaHCO_3_: 0 mmol L^-1^ (control) for the CK treatment, and 25 mmol L^-1^ (T1), 50 mmol L^-1^ (T2), 75 mmol L^-1^ (T3), and 100 mmol L^-1^ (T4) for the experimental treatments. The mixed solution was added gradually to prevent osmotic damage to the seedlings, and the treatment concentrations were replaced with 25 mmol L^-1^ solutions daily until the preset concentration was attained. The salt treatments were watered to maintain field capacity. In accordance with these criteria, the seedlings were watered every day until a steady state was reached. On the 30th day after the salt treatment was completed, plants in each treatment were randomly selected for follow-up tests.

### Leaf water content

Two to four fresh leaves were weighed and denoted as W1. They were put in an oven at 105°C and transferred to an 80°C environment to dry for 2 h, and then the dry mass was weighed (W2). Finally, the leaf water content was calculated: Leaf water content (%) = [(W1-W2)/W1] × 100 (Zhou et al., 2021).

### Malondialdehyde and relative conductivity

A 0.5 g sample of fresh leaves was homogenized in 0.05 mol L^-1^ trichloroacetic acid, after which the homogenate was centrifuged at 5, 200 rpm for 20 min. Two mL of the supernatant and 2 mL of thiobarbituric acid were incubated together in a water bath (100°C, 30 min). The cooled mixture was centrifuged, and the absorbance of the supernatant was measured at 450, 532, and 600 nm by a spectrophotometer. The content of MDA was determined (Li, 2000). Between 15 and 25 leaf samples cut out with around hole puncher were placed in 10 mL of distilled water for 4 hours, and then electrical conductivity was measured with an EC meter and recorded as EC1. The samples were incubated in a water bath (100°C) for 15 min and the electrical conductivity was re-measured (EC2). The electrolyte leakage percentage was calculated based on a pre-existing equation (Li, 2000).

### Proline content and soluble sugar content

A 0.5 g fresh sample of plant material was freeze-dried and then had to be powdered by liquid nitrogen, and then extracted with 5 mL of 3% sulfosalicylic acid in a water bath (100°C) for 10 min. The homogenates were subsequently filtered and 2 mL of solution after filtration were heated with 2 mL of glacial acetic acid and 2 mL 2.5% acid-ninhydrin for 30 min at a temperature of 100°C. Then the cooled filtrates were immersed in 4 mL of toluene and vibrated. The extract was then centrifuged at 3000 rpm for 5 min. Finally, the sample absorbance was determined at 520 nm by spectrophotometry. Soluble sugar was extracted as follows: 0.05 g of fresh leaf was homogenized with 10 mL distilled water in a mortar and pestle. After heating the homogenate in a water bath (60°C) for 30 min, the homogenate was centrifuged at 5000 rpm for 5 min. The 0.5 mL supernatant was mixed with 1.5 mL distilled water, 0.5 mL ethylanthrone acetate, and 5 mL concentrated sulfuric acid. It was then shaken quickly and well, and the sample absorbance was determined at 620 nm by spectrophotometry. The proline and soluble sugar content were calculated using a formula (Li, 2000).

### Assays of antioxidant enzyme activity

A 0.5 g sample of fresh leaves was homogenized in 5 mL of 0.05 mol L^-1^ phosphate buffer (1 mmol L^-1^ EDTA, 1% PVP, pH 7.8), and then the homogenate was centrifuged at 12,000 rpm for 20 min at 4°C. The supernatant was then used to determine antioxidant enzyme activity. The activities of superoxide dismutase (SOD), catalase (CAT) and peroxidase (POD) were determined by the UV absorption method (Shi et al., 2011)

### Chlorophyll fluorescence parameters

The Li-6400 portable photosynthesis system with chlorophyll fluorescence leaf chamber (Li-Cor, Inc., Lincoln, NE, USA) was used to measure the chlorophyll fluorescence parameters. After 20 minutes of dark adaptation, the fluorescence parameters of the middle and upper functional leaves were measured and calculated. Parameters such as the initial fluorescence (*F_o_*), maximum fluorescence (*F_m_*), steady-state fluorescence (*F_s_*), maximum fluorescence signal (*F_m_’*), and minimum fluorescence (*F_o_’*) can be directly measured by the instrument. The relevant indicators were calculated: variable fluorescence (*F_v_*) =*F_m_*- *F_o_*; Potential activity of PS II (*F_v_*/*F_o_*) = (*F_m_*-*F_o_*)/*F_o_*; Potential photochemical efficiency of PSII (*F_v_*/*F_m_*) = (*F_m_*-*F_o_*)/*F_m_*; Photosynthetic electron transfer efficiency (ETR) = [(*F_m_’*-*F_s_*)/*F_m_’*] ×PFD×0.5×0.84; PS II actual photochemical efficiency (Yield) = (*F_m_’*- *F_s_*)/*F_m_’*.

### Data analysis

All the data are expressed as the mean and standard deviation of three experimental replicates. All statistical analyses were performed using SPSS version 22.0 (IBM, Armonk, N.Y., USA). Significance testing was done using one-way analysis of variance (ANOVA) and Duncan’s multiple range tests (*p* < 0.05). Charts were plotted using Microsoft Office Excel 2016.

## Results

### Salt injury symptoms and leaf water content of two maple species under salt stress

**Figure 1:**
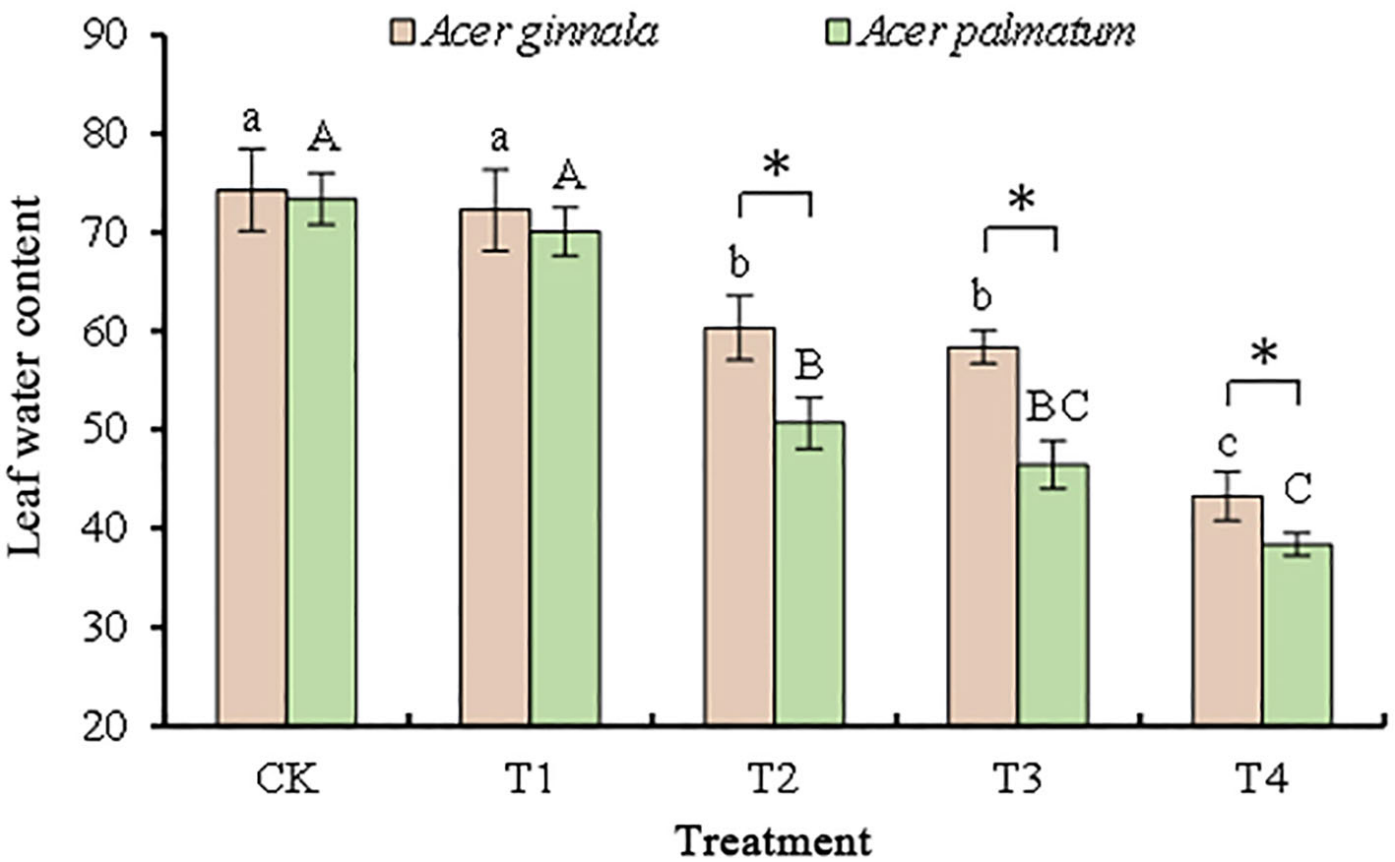
Effects on the leaf water content under different salinity levels in *Acer s*eedling. Data are the mean ± SD. Significant differences at *p* < 0.05 (Duncan’s test) of *Acer ginnala* and *Acer palmatum* were expressed by different small letters and capital letters, respectively. * refer to significant differences at 0.05 level between the two maples.

The salt injury symptoms were investigated, and the leaf morphology did not change after the 25 mmol L^-1^ salt stress trial. About 20%–30% of the leaf tips and margins of the seedlings showed injury symptoms such as water loss and wilting after the 50 mmol L^-1^ salt stress trial. With an increase in the salt concentration, the degree of leaf salt damage gradually increased (*p* < 0.05). More than 70% of the leaf tips and leaf margins of the seedlings showed severe injury symptoms such as water loss, wilting and dry rot of leaves after receiving 100 mmol L^-1^ salt stress. The leaf water content of the two maple tree species exhibited a similar trend. At 25 mmol L^-1^ salt stress, there was no significant difference in leaf water content compared to the control. However, at concentrations greater than 50 mmol L^-1^, the leaf water content was significantly lower than the control, especially at 100 mmol L^-1^ salt stress, where the leaf water content decreased by 45%-50% (**Figure 1**). These results indicated that different salt concentrations caused different degrees of damage to the leaves of *Acer ginnala* and *Acer palmatum*.

### Effects on cell membrane permeability in leaves under salt stress

**Figure 2:**
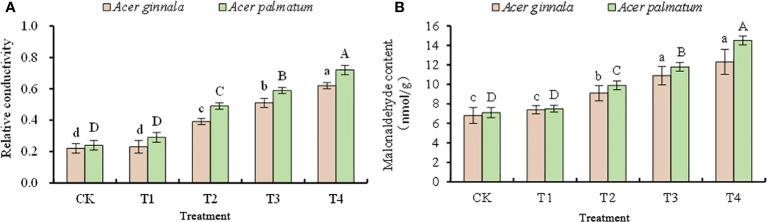
Effects on the relative conductivity **(A)** and malonaldehyde content **(B)** under different salinity levels in *Acer s*eedling. Data are the mean ± SD. Significant differences at *p* < 0.05 (Duncan’s test) of *Acer ginnala* and *Acer palmatum* were expressed by different small letters and capital letters, respectively.

With an increase in the NaCl and NaHCO_3_ solutions, the relative conductivity and malondialdehyde content of the leaves showed an increasing trend, but the range of increase was different (**Figure 2**). Exposure to 25 mmol L^-1^ NaCl and NaCO_3_ mixed solution resulted in no statistical difference in the relative conductivity and MDA content between the four treatments and the control (*p* > 0.05); treatments of NaCl and NaHCO_3_ at concentrations higher than 50 mmol L^-1^ resulted in higher relative conductivity and MDA contents than the control (*p* < 0.05), which indicates that salt stress caused damage to the cell membrane of the two species. In particular, when the concentration of the NaCl and NaHCO_3_ mixture was 100 mmol L^-1^, the relative conductivity of *Acer ginnala* and *Acer palmatum* increased by 1.81-fold and 2.0-fold, respectively, compared with the control, and the content of MDA increased by 80.9% and 104%, respectively. The above results show that the increased amplitude for *Acer ginnala* was lower than that for *Acer palmatum* (*p* < 0.05), indicating that the membrane damage of *Acer ginnala* caused by high salt concentration was significantly less than that of *Acer palmatum*.

### Effect on osmotic regulators in leaves under salt stress

**Figure 3:**
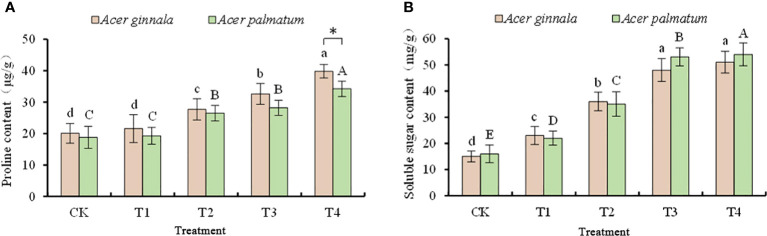
Effects on proline content **(A)** and soluble sugar content **(B)** under different salinity levels in *Acer s*eedling. Data are the mean ± SD. Significant differences at *p* < 0.05 (Duncan’s test) of *Acer ginnala* and *Acer palmatum* were expressed by different small letters and capital letters, respectively. * refer to significant differences at 0.05 level between the two maples.

The accumulation of proline in maple leaves was not increased in the 25 mmol L^-1^ treatment (*p* > 0.05), and the proline content in other treatments was higher than that of the control (*p* < 0.05, **Figure 3**). When the concentration of salt stress reached 100 mmol L^-1^, the proline content of *Acer ginnala* and *Acer palmatum* increased by 98.0% and 81.9%, respectively, compared with the control. an increase in the concentration of NaCl and NaHCO_3_, the soluble sugar content of the two species showed a gradually increasing trend, and the soluble sugar content of each treatment was higher than that of the control (*p* < 0.05). In the 100 mmol L^-1^ salt stress, the soluble sugar contents of *Acer ginnala* and *Acer palmatum* leaves were 2.4-fold and 2.38-fold of the control, respectively. In conclusion, the leaves of both species increased their osmotic potential and alleviated osmotic stress by accumulating soluble sugar and proline under salt stress.

### Effects on antioxidant enzyme activities in leaves under salt stress

**Figure 4:**
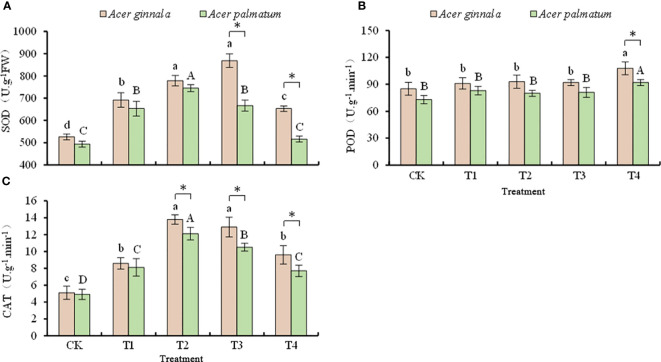
Effects on the activity of SOD **(A)**, POD **(B)**, and CAT **(C)** under different salinity levels in *Acer s*eedling. Data are the mean ± SD. Significant differences at *p* < 0.05 (Duncan’s test) of *Acer ginnala* and *Acer palmatum* were expressed by different small letters and capital letters, respectively. * refer to significant differences at 0.05 level between the two maples.

The antioxidant enzyme activities under salt stress are shown in **Figure 4**. At a NaCl and NaHCO_3_ solution concentration of 75 mmol L^-1^, the SOD activity of *Acer ginnala* reached the maximum (65.2% higher than the control). At a NaCl and NaHCO_3_ concentration of 50 mmol L^-1^, the SOD activity of *Acer palmatum* reached the maximum (51.1% higher than the control). At a NaCl and NaHCO_3_ solution concentration higher than 75 mmol L^-1^, *Acer ginnala* had higher SOD activity than *Acer palmatum* (*p* < 0.05).

The POD activity of *Acer ginnala* and *Acer palmatum* increased by 27.1% and 26.0%, respectively, only when the NaCl and NaHCO_3_ solution concentration was 100 mmol L^-1^; there was no statistical difference in POD activity between the other treatments and the control (*p* > 0.05).

With an increase in the salt concentration, the CAT activity of both species had the same change trends of first increasing and then decreasing. When the concentration of the NaCl and NaHCO_3_ solution was 50 mmol L^-1^, the CAT activity of *Acer ginnala* and *Acer palmatum* reached the maximum, which increased by 170% and 147% compared with the control, respectively; the increased range of *Acer ginnala* was greater than that of *Acer palmatum* (*p* < 0.05). The CAT activity of both species gradually decreased with an increase in the salt concentration (*p* < 0.05). In response to the 50, 75, and 100 mmol L^-1^ NaCl and NaHCO_3_ solution treatments, *Acer ginnala* had higher CAT activity than *Acer palmatum* (*p* < 0.05).

### Effects on the chlorophyll fluorescence parameters in leaves under salt stress

**Figure 5:**
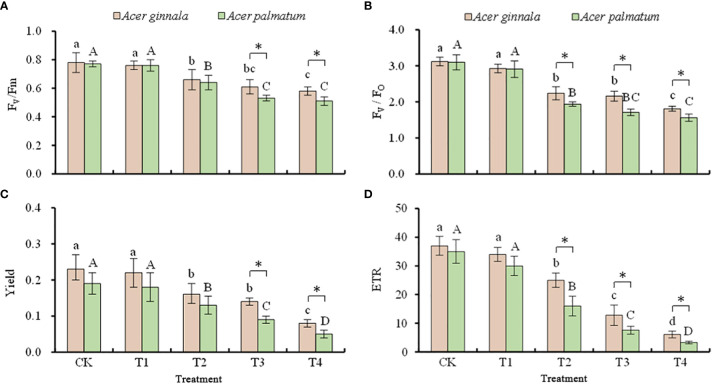
Effects on the F_v_/F_m_ **(A)**, F_v_/F_o_ **(B)**, Yield **(C)**, and ETR **(D)** values under different salinity levels in *Acer* seedling. Data are the mean ± SD. Significant differences at *p* < 0.05 (Duncan’s test) of *Acer ginnala* and *Acer palmatum* were expressed by different small letters and capital letters, respectively. * refer to significant differences at 0.05 level between the two maples.

The effects of different concentrations of NaCl and NaHCO_3_ solutions on the chlorophyll fluorescence parameters of both maple species are shown in **Figure 5**. When the concentration of the NaCl and NaHCO_3_ solution was 25 mmol L^-1^, there were no changes in the fluorescence parameters of both *Acer* seedlings (*p* > 0.05), which indicates that salt stress lower than 25 mmol L^-1^ had little effect on leaf PSII. The *F_v_*/*F_m_*, *F_v_*/*F_o_*, Yield and the ETR of both species gradually decreased with an increase in the salt concentration (*p* < 0.05). Compared with the control, when the solution concentration of NaCl and NaHCO_3_ was 100 mmol L^-1^, the *F_v_*/*F_m_*, *F_v_*/*F_o_*, Yield, and ETR of *Acer ginnala* leaves decreased by 25.6%, 41.9%, 65.2%, and 83.5%, respectively, while the *F_v_*/*F_m_*, *F_v_*/*F_o_*, Yield, and ETR of *Acer palmatum* decreased by 33.7%, 49.7%, 73.7%, and 90.6%, respectively. The results showed that the decreasing range of fluorescence parameters in *Acer ginnala* leaves was smaller than that of *Acer palmatum*, indicating that the effect of salt stress on PSII in *Acer ginnala* leaves was relatively smaller than that of *Acer palmatum*.

## Discussion

Previous related research on other plant species has demonstrated that under low-concentration salt stress, the leaf relative water content remains relatively unchanged, and the leaves do not exhibit symptoms of salt damage, In contrast, under high-concentration salt stress, the water content is significantly reduced, resulting in severe leaf dehydration (Hernández and Almansa, 2002). In our study, we observed a decrease in leaf water content starting at 50mmol L^-1^ NaCl and NaHCO_3_ stress, with a notable decrease to 40% at a concentration of 100 mmol L^-1^. The high salt ion concentrations disrupt the ion balance and induce water deficit, which is consistent with similar findings in *Quercus dentata* (Hao et al., 2020).

High concentrations of salt can cause ion toxicity, membrane system destruction, and an increase in membrane permeability, resulting in an increase in the relative conductivity of plants (Esfandiari et al., 2020; Zhang et al., 2020). An increased relative conductivity can reflect the degree of damage caused to plant cell membranes under adverse conditions, and a relative conductivity of greater than 50% will cause fatal damage to the cell membrane (Lu et al., 2015). In this study, the relative conductivity of the two maple species reached 50% under a salt solution concentration of 75 mmol L^-1^, indicating that the cell membranes were severely damaged. MDA is also an important physiological indicator that reflects the integrity of cell membranes. A change in the MDA content can reflect the extent of environmental damage to plants and the severity of plant responses to environmental stimuli (Lang et al., 2020; Zhang et al., 2020). Our study shows that with an increase in salt concentrations, the MDA content of both maple species gradually increased, and the MDA increase of *Acer ginnala* was relatively lower than that of *Acer palmatum*. This indicates that under salt stress, there was a small change in the cell membrane permeability and a low degree of peroxidation of the cell membrane of *Acer ginnal. Acer ginnala* may be better able to maintain cell membrane integrity and the stability of the membrane structure, thus alleviating the oxidative damage caused by salt stress to the cells.

In order to adapt to a high saline environment, plants can synthesize organic solutes to adjust their intracellular osmotic potential and to maintain water balance (Han et al., 2020). Soluble sugar not only participates in osmotic regulation but also provides a carbon framework and energy for the synthesis of other organic compounds in plants. It also plays an important role in the protection of various enzymes in cells and the stability of membrane systems (Heuer, 2003). In this study, salt stress increased the soluble sugar content; similar results were found for *Magnolia biondii* (Shen et al., 2021) and *Chimonanthus praecox* (Li et al., 2021). The soluble sugar content of some salt-tolerant plants increased at first and then showed a downward trend under high-concentration salt stress, which may be due to the fact that high-concentration salt stress requires more energy allocation to maintain resistance (Zhang et al., 2020). Our study did not show such results, which may be related to the salt tolerance of maples in general.

Under salinity stress, a high accumulation of proline plays an important role in cell adaptations to salt by increasing the acidity of the cytoplasm and maintaining the ratio of NADP^+^ to NADPH (Esfandiari et al., 2020). One study found that salt stress activated proline synthetase in *Malus micromalus* and inhibited the activity of proline-degrading enzymes, which led to the accumulation of proline (Hu et al., 2020). Proline accumulation in plants has been found to positively correlate with salt tolerance (Elik and Atak, 2012). In our study, the free proline content of both maple species gradually increased with an increase in the salt concentration, and under high salt stress, *Acer ginnala* accumulated more proline, indicating that this species regulates the microenvironment by accumulating proline, thereby enhancing its adaptability to a salt-treated environment.

Under no-stress circumstances, the reactive oxygen species (ROS) produced in plant cells can be eliminated by the antioxidant protection system and will not cause damage to plants (Hasanuzzaman et al., 2021). However, salt stress can cause the accumulation of ROS in plants and can cause oxidative damage to plant cells (Mudgal et al., 2010). To mitigate oxidative damage induced by excessive ROS, plants have developed a complex defense antioxidative system (Zhang et al., 2012). Antioxidant enzyme systems, including SOD, CAT, and POD, are important systems for removing ROS and play an important role in maintaining metabolic balance in plants (Neto et al., 2006). Among these, SOD first converts superoxide O_2_^−^ into O_2_ and H_2_O_2_. Then, CAT and/or POD converts H_2_O_2_ into H_2_O and O_2_ to reduce the toxicity of H_2_O_2_ in plant cells (Apel and Hirt, 2004). Previous studies have shown that salt stress increases SOD and CAT activities in *Lycopersicon esculentum* seedlings (Gong et al., 2013) as well as SOD, CAT, and POD activities in *Cucumis sativus* (Tang et al., 2018). This suggests that salt stress can alter the activities of enzymes involved in scavenging ROS in various species, and these ROS-scavenging enzymes are co-regulated in plants. In our study, the SOD and CAT activities of the two maple species first increased and then decreased, while the range of the change in POD activity was small and was only significantly higher than that in the control when the NaCl and NaHCO_3_ solution concentration was 100 mmol L^-1^. We also showed that under salt stress, the antioxidant capacity was enhanced by a collaboration of SOD and CAT in order to remove excess ROS and reduce damage to the cell membranes. In particular, *Acer ginnala* had more SOD and CAT activity than *Acer palmatum* under different concentration of salt stress, which shows that *Acer ginnala* may metabolize ROS faster than *Acer palmatum* by maintaining relatively high SOD and CAT activities. Similar results were found for *Quercus dentata* (Hao et al., 2020). However, the activity and gene expression of SOD, POD, CAT, and APX in *Cucumis sativus* seedlings were significantly increased by salt stress (Tang et al., 2018). In response to NaHCO_3_ stress, the activity and expression of CAT in *Morus alba* seedling leaves were significantly decreased, but the H_2_O_2_ scavenging ability of POD was enhanced (Zhang et al., 2020). In our study, both maple species mainly relied on the synergistic action of SOD and CAT enzymes to remove active oxygen to alleviate the lipid peroxidation damage of cell membranes.

Any influence of environmental factors on photosynthesis can be reflected through changes in chlorophyll fluorescence parameters (Alvarez-Acosta et al., 2018). Numerous studies have shown that chlorophyll fluorescence parameters are very sensitive indices that can be used to indicate the photosynthetic physiological status of plants and to evaluate the health status under stressful conditions (Gao et al., 2021). The *F_v_*/*F_m_* ratio is an index of the maximum photochemical efficiency of PSII, and a decrease in this parameter is a reliable sign of photoinhibition (Gong et al., 2013; Miao et al., 2020). In our study, the *F_v_*/*F_m_* of the leaves of both species gradually decreased with an increase in salt concentration, which showed they produced photoinhibition substances. The Yield and ETR also exhibited a significant downward trend with an increase in the salt concentration, and the decrease under high salt stress was particularly notable. Similar findings were reported in a study on *Malus robusta* (Hu et al., 2020). Our results indicate that under high salt conditions, the actual photosynthetic quantum yield and apparent synthetic electron transfer efficiency of maple leaves were inhibited. However, the decline in the fluorescence parameters of *Acer ginnala* was smaller than that of *Acer palmatum*, suggesting that salt stress had a relatively lesser impact on the PSII of *Acer ginnal.*

Our study primarily focused on the physiological responses of two maple species to salt stress. However, enhancing the salt tolerance of maple trees remains a challenge. Several approaches, such as supplementary putrescine (Esfandiari et al., 2020) salicylic acid (Miao et al., 2020), methyl jasmonate (Lang et al., 2020), melatonin (Zhang et al., 2020), nitrogen fertilization (Duan and Chang, 2017), and potassium and calcium (Zrig et al., 2021), have been employed to enhance salt stress tolerance in other plant species. To expand the range of landscaping options for saline areas, further research is necessary to explore methods for improving the salt tolerance of maple trees.

## Conclusion

Our results demonstrate that different degrees of mixed salt stress have different impacts on physiological characteristics. Under moderate or high saline-alkali stress, the relative conductivity and malondialdehyde content significantly increased, while the chlorophyll fluorescence parameters *F_v_*/*F_o_*, *F_v_*/*F_m_*, Yield, and ETR decreased. Both maple species adjusted their osmotic balance and alleviated oxidative stress by accumulating proline and soluble sugars, as well as increasing the activities of CAT and SOD. It can be observed that both maple species exhibit a certain level of salt tolerance, with *Acer ginnala* demonstrating better salt tolerance compared to *Acer palmatum*, Therefore, they can be utilized for greening purposes in low-saline soil.

## Data availability statement

The original contributions presented in the study are included in the article/supplementary material. Further inquiries can be directed to the corresponding authors.

## Author contributions

LR and ZZ conceived this project. YG and BX designed experiments and interpreted the results. LC, XL, XZ, XW, YW, BW, WZ, and CL performed the experiments and analyzed the data. All authors contributed to the article and approved the submitted version.
